# Why Not a Sound Postulate?

**DOI:** 10.1007/s10701-021-00479-0

**Published:** 2021-06-21

**Authors:** Bryan Cheng, James Read

**Affiliations:** University of Oxford, Oxford, UK

**Keywords:** Special relativity, Sound postulate, Waves, Lorentz invariance, Analogue gravity

## Abstract

What, if anything, would be wrong with replacing the light postulate in Einstein’s 1905 formulation of special relativity with a ‘sound postulate’, stating that the speed of sound is independent of the speed of the source? After reviewing the historical reasons underlying the particular focus on light in the special theory, we consider the circumstances under which such a theory of ‘sonic relativity’ would be justified on empirical grounds. We then consider the philosophical upshots of ‘sonic relativity’ for four contemporary areas of investigation in the philosophy of spacetime: (i) global versus subsystem symmetries, (ii) dynamical versus geometrical approaches to spacetime, (iii) the possibility of a preferred frame in theories of quantum gravity, and (iv) spacetime functionalism.

## Introduction

Suppose that one is modelling the propagation of a sound wave, $\psi \left( t, x^i\right)$. As with other waves, its evolution is described by the wave equation,

1 $$\begin{aligned} \frac{ \partial^2 \psi }{\partial t^2} = u^2 \frac{\partial^2 \psi }{\partial {x^i}^2}, \end{aligned}$$

where *u* is the velocity of the wave through its medium in the rest frame of that medium; in the case of a sound wave, the medium is the air (or water, or any other medium in which sound waves can propagate—for simplicity, we will without loss of generality speak of the medium of sound waves as being the air in what follows). Since (1) is a massless Klein–Gordon equation, it has the symmetries of that equation^1^: for our purposes, the important class of such symmetries to note are the Poincaré transformations, though with invariant speed given by *u*.^2^ (For a derivation of this result, see e.g. [28].) In other words, the propagation of sound waves is modelled in the same manner as the propagation of (say) electromagnetic waves (wave equations for which can be derived from Maxwell’s equations—see e.g. [32]), insofar as both are governed by Poincaré invariant wave equations: the only difference between these symmetries is the invariant speed (which, naturally, is *c* in the case of light).^3^

The wave equation for sound, as a description of a vibrating string, has been known since at least the seminal 1746 work of d’Alembert [17, p. 354]; moreover, the parallels between the descriptions of the propagation of light and sound were well-appreciated by the nineteenth Century.^4^ Given that the wave equation for sound is Poincaré invariant, one ignorant of the relevant background might be prompted to ask the following historical question: why did Einstein focus upon light in his 1905 presentation of special relativity [22], when other Poincaré invariant physics was also known at that time? Put in other words, when one considers Einstein’s two postulates of special relativity—

- *Relativity principle* The laws by which the states of physical systems undergo change are not affected, whether these changes be referred to the one or the other of two systems of coordinates in uniform translatory motion.
- *Light postulate* Any ray of light moves in the ‘stationary’ system of coordinates with the determined velocity *c*, whether the ray be emitted by a stationary or by a moving body. Hence
  $$\begin{aligned} \text {velocity} = \frac{\text {light path}}{\text {time interval}}. \end{aligned}$$
  (Here, ‘time interval’ is to be understood in the sense articulated in [22, Sect. 1].)

—one can ask: what, if anything, would have been wrong with replacing the latter of these two principles with the following?:

- *Sound postulate* Any ray of sound moves in the ‘stationary’ system of coordinates with the determined velocity *u*, whether the ray be emitted by a stationary or by a moving body. Hence
  $$\begin{aligned} \text {velocity} = \frac{\text {sound path}}{\text {time interval}}. \end{aligned}$$
  (Here, ‘time interval’ is to be understood in the sense articulated in [22, Sect. 1].)

The speed of sound through a medium is, indeed, independent of the speed of the source: it is a function of the velocity of the medium in which the sound propagates. This is what gives rise to the familiar physics of e.g. sonic booms in supersonic travel. So: prima facie, the parallels between light and sound run deep. Why, then, Einstein’s focus on light in the 1905 paper?

The correct answer to this question—the answer which we will elaborate in detail over the course of this paper—is that, for any medium capable of transmitting sound, there are phenomena which depend on motion with respect to that medium. Thus, the sonic Poincaré symmetries in the acoustic case are broken when sound waves interact with other physical systems; this symmetry breaking manifests in empirically detectable effects (again, such as sonic booms). By contrast, there are no known phenomena which depend on motion with respect to putative medium of light—the so-called ‘luminiferous ether’. Thus, there are empirical grounds—accrued with painstaking care over the course of the nineteenth century—for believing in the existence of a sound medium, but not for believing in the existence of the ether; in this sense, light is ‘fundamental’, but sound is not (we will clarify this notion of fundamentality, and its relation to others, in the body of this paper). Having for this reason (based upon application of Occam’s razor) rejected belief in the existence of this ether, one can then understand the 1905 special theory as constituting the inductive base for a universal kinematical constraint that *all* physical fields without media (in the case of light: the ether) should obey Poincaré invariant physical laws, with invariant speed given by *c*.

Articulating this account in full detail and rigour is the central goal of this paper. Our plan is the following. We begin in Sect. 2 with historical considerations regarding the genealogy of the focus upon light, rather than upon other waves described by Poincaré invariant equations (such as sound), in the development of special relativity. Having clarified these matters, we turn in Sect. 3 to the question of how much of special relativity one could recover by replacing Einstein’s light postulate with a sound postulate, and under what circumstances such as theory of ‘sonic relativity’ would be justified on empirical grounds; here, we take ourselves to be providing a philosophical compliment to the recent beautiful technical results on these matters to be found in [5, 41, 61, 62].^5^ In Sect. 4, we consider how our conclusions presented in the previous sections bear on the case of gravitational waves. Finally, in Sect. 5 we explore some upshots of our work vis-à-vis four ongoing areas of investigation in the philosophy of spacetime and symmetries: (i) global versus subsystem symmetries, (ii) dynamical versus geometrical approaches to spacetime, (iii) the possibility of a preferred frame in theories of quantum gravity, and (iv) spacetime functionalism.

## History: The Special Role of Light

Consider Galileo’s famous ship thought experiment: in the first scenario, a ship is stationary with respect to the shore; in the second, the ship is moving uniformly with respect to the shore, with the motion “not fluctuating this and that” [27]. When considering physical processes inside the ship, Galileo notes that “you will discover not the least change in all the effects named”: this is one expression of the relativity principle, taken to hold with respect to the Galilean boosts (i.e., uniform velocity boosts of the form $x \mapsto x' = x - vt$). Do sound waves satisfy this Galilean relativity principle? In answering this question, there are two cases to analyse: (a) a dragged medium, and (b) a stationary medium.

First, consider a sound wave propagating in the interior of Galileo’s ship. Assuming that the sound medium (i.e., the air inside the ship) is also subject to the uniform velocity boost (which, in this case, it is!), the answer to the above question is *yes*: the propagation of the sound will proceed in exactly the same manner inside the ship in both cases, for in both cases is the observer co-moving with the medium in which the wave is propagating. Thus: *if the medium of propagation is also subject to a Galilean boost, one does not expect violations of the Galilean relativity principle by a given wave*.

Contrast this with a second example: an aeroplane emitting sound from its engines as it travels through the air. More specifically: consider two otherwise-identical planes, moving at different velocities through the air. In this case, the medium (the air) is not subject to a Galilean boost (which relates the two planes); since the velocity of the sound emitted by the planes is a function only of the speed of the medium (here the air, assumed to be at rest), and (as with all waves) not of the speed of the source (the plane’s engines),^6^ this leads to the sound propagating at different velocities with respect to the plane in the two cases—which can, in turn, lead to empirically distinguishable effects (even from within the plane), e.g. sonic booms. Thus: *if the medium of propagation is not also subject to a Galilean boost, one does in general expect violations of the Galilean relativity principle by a given wave*.

Thus, if the medium is dragged along with the subsystem, as with Galileo’s ship, then Galilean symmetries will not be broken. Yet even when the medium is not dragged, and observers’ moving through a stationary medium causes Galilean symmetry-breaking effects, one might reasonably argue that these effects are simply a peculiarity of the easily-detectable and tangible medium of sound, rather than a fundamental issue with Galilean symmetries as a universal constraint.

Now, in their investigations into light and electromagnetism in the nineteenth century, physicists made the (quite reasonable) assumption that, just as sound waves propagate in a medium (the air), so too should light waves propagate in a medium: this medium came to be known as the ‘luminiferous ether’.^7^ If the ether were indeed dragged by moving bodies, then one would not expect violations of the Galilean relativity principle, on Galilean boosts—exactly analogous to the case of sound on Galileo’s ship.^8^ Given the null result of first order experiments such as Arago’s measurements of stellar aberration, it was hypothesised (most significantly by Fresnel) that the Earth at least partly drags the luminiferous ether as it moves around the Sun: in this way, physicists could account for there being no *detectable* difference in the results of electromagnetic experiments at different times of the year [18, 54].

As Ryckman puts it, though, “the idea that a body moving through a stationary ether partially drags along both the ether and the light propagating through it with a tiny fraction of the body’s velocity added yet another mysterious property to the ethereal substance” [54, pp. 172–173]. Furthermore, the null result of the Michelson–Morley experiment, which was the only successful second order experiment conducted, cast doubt on Fresnel’s account, which had predicted detectable effects from ether currents at second order accuracy [45, Chap. 8]. Thus, physicists began exploring the possibility of simply dropping the ether drag hypothesis. The consequence, of course, was a failure of the Galilean relativity principle: if the ether is not dragged by the motion of the Earth, then, just as in the case of the aeroplane described above, one would expect experimentally distinguishable effects, depending upon the Earth’s state of motion. In order to restore the apparent empirical *in*distinguishability of the different states of the Earth’s motion around the Sun (effectively, empirical indistinguishability of different states of the Earth’s motion under Galilean boosts), physicists such as Lorentz and FitzGerald instead introduced dynamical contraction hypotheses: the physics governing the constitution of material bodies must (they hypothesised) cause them to contract in just such a way as to counteract the effects from the non-dragging of the ether, and lead, fortuitously, to no *detectable* differences under Galilean boosts of the Earth; of course, such hypotheses also faced the spectre of ad hocness.^9^

The foregoing well-known history goes a long way to accounting for Einstein’s particular focus on light in his 1905 paper on special relativity: given that, by the end of the nineteenth century, physicists had convinced themselves that the ether (unlike the air in Galileo’s ship) does *not* drag with the Earth’s motion, it was the particular nature of (the medium of propagation of) light which seemed to raise problems for the Galilean relativity principle.^10^ Moreover, unlike sound (as in the above example of the plane), the particular state of motion of this medium of propagation could not (it seemed) be ascertained except via reconstruction from observed Galilean relativity principle violating effects (as per the Michelson–Morley experiment: effects which were never, in fact, observed). And finally: light and its medium were taken by many later 19th Century ether theorists to differ fundamentally from other forms of waves, such as sound or water, insofar as the light medium was thought not to be reducible to more familiar matter (e.g. a lattice of known molecules).^11^ It was the fact that the medium of light propagation was (a) hypothesised never to be dragged, (b) intangible in a way that other media of propagation (say air, in the case of sound) were not, and (c) apparently of an entirely distinct nature to that of other physical entities, which meant that it was light in particular which cried out for reconciliation with the Galilean relativity principle—for many of the physical explanations which served to save this principle in other contexts (such as in the above example regarding the plane) were, given the above points, not readily applicable to the case of light.

Now, there are open questions regarding whether, with the completed theory of special relativity in hand, one need say that light plays a crucial role in this theory—or rather, just as with the equivalence principle of general relativity, it played the role only of ‘midwife’ in the construction of the theory.^12^ Later in his life, for example, Einstein wrote this:

The special theory of relativity grew out of the Maxwell electromagnetic equations. But ... the Lorentz transformation, the real basis of special-relativity theory, in itself has nothing to do with the Maxwell theory. [23]

Rather, in the view of the later Einstein, the situation was this:

The content of the restricted relativity theory can accordingly be summarised in one sentence: all natural laws must be so conditioned that they are covariant with respect to Lorentz transformations. [24]

Notwithstanding his reneging on the focus of light, though, and his later understanding of the role of special relativity as simply issuing a universal kinematical constraint of Poincaré invariance,^13^ the above considerations help us to understand the historical precedent for Einstein’s focusing on light in his 1905 paper, in spite of it being well-known that other waves, such as sound, obey exactly the same dynamical equations as electromagnetic waves. With these historical considerations in hand, we can now address a more conceptual question: to what extent *could* one arrive at the special theory of relativity using a sound postulate?

## Contemporary Considerations

Suppose that one is in a laboratory, and that one is able to perform experiments using sound, but not using light. Moreover, suppose that there are (e.g.) no air conditioners, so that the air is stationary in the laboratory. In this situation, one could construct, *within the laboratory*, Galileo-ship-type experiments; since the air is at rest in the lab, one would, within these individual experimental setups (now themselves within the laboratory), expect violations of Galilean relativity principles. If such violations were not observed, one could expect the history of special relativity to play out once more, but now with respect to sonic experiments in the lab: one might hypothesise either (a) that the air is ‘dragged’ after all, or (b) that there is some principle of ‘sonic dynamical contraction’ of the experimental setups to cancel the relativity principle-violating effects, or (c) (as *per* Einstein in 1905) that the entire hypothesis of a sound medium is redundant, and that one should understand sonic Poincaré invariance as a universal kinematical constraint.

Suppose one went for option (c) here. Then, mirroring Einstein in 1905, one could construct a theory of ‘sonic relativity’, based on the relativity principle and the sound postulate (cf. Sect. 1).^14^ Indeed, it has recently been shown, in elaborate and quite beautiful detail, that by constructing in a lab familiar special relativistic objects such as Langevin clocks, but using sound as a surrogate for light,^15^ one would be able to derive in this way all familiar special relativistic effects, such as length contraction, time dilation, and the relativity of simultaneity [61].^16^

In a certain sense, then, if one were to be asked the question: ‘What is wrong with formulating the special theory of relativity on the basis of a sound postulate?’, one might be inclined—at least within the context of the ‘sonic laboratory’—to answer as follows: *nothing*. This can’t, however, be the whole story. There must be some deeper reason why sound cannot be an appropriate base upon which to ground special relativistic physics.

The difference between light and sound in this regard, as we see it, is a matter of fundamentality. Here, by the ‘fundamentality’ of a theory of waves, we mean this: the theory describes waves which are understood to be self-subsistent, rather than to be oscillations in some medium.^17^ In this sense, sound waves are not fundamental, as they can be understood as higher-level descriptions of oscillations in some antecedently-given ontology—viz., the air. By contrast, electromagnetic waves are fundamental, for they are not to be understood as higher-level descriptions of oscillations in some antecedently-given ontology. For example, when one writes down a wave solution $A^\mu = \xi^\mu e^{i S}$ (the wave vector being given by $k^\mu = \partial^\mu S$), one understands that the electromagnetic field *just is* the wave: unlike air in the case of sound, the electromagnetic field here is not ontologically prior to the wave.^18^ This different understanding of sound versus light vis-à-vis fundamentality in our sense is grounded in empirical results: as we have already seen, for media capable of transmitting sound, there are phenomena that depend on motion with respect to that medium; not so for light.

Why do we connect the fundamentality of waves with non-dependence on media? On the one hand, if a wave has a medium, then that medium must necessarily consist of *something*,^19^ and so the wave is not *per se* fundamental. On the other hand, if a wave is not fundamental, then either (i) it is reducible to oscillations in some medium, or (ii) it can be associated with a self-subsistent entity (say the electromagnetic field) which can in itself be reduced to some more fundamental physical entity.^20^ Setting aside in this section the (entirely plausible) second possibility of reduction to a more fundamental entity (we return to this understanding of the notion in Sect. 5.3), there is a natural coincidence between a wave being fundamental and its not depending on a medium.

Now, post-Einsteinian theories of light and electromagnetism (viz., classical and quantum electrodynamics) are fundamental theories in this sense—unlike theories of sound propagation. This is significant, for, as already mentioned, the import of special relativity (*qua* kinematical constraint) is supposed to apply not just to electromagnetism, but to *all* fundamental physics (where ‘fundamental’ is still to be understood as above). That is, Einstein’s 1905 paper can be understood as involving an implicit inductive extrapolation, from the Poincaré invariance of the laws governing light (one kind of fundamental physical field), to the Poincaré invariance of all fundamental physical fields. It is not at all clear that this extrapolation (which could be construed in various ways—for example, either as straightforwardly inductive, or as involving a shift in Friedman’s ‘relativised a priori’ [26]) would be warranted were it to be based upon a field *not* regarded as being fundamental—for in this case there would be *no* inductive base on which to form this extrapolation about all fundamental physics; moreover, the dependence of empirical phenomena upon the velocity of the sound medium warrants the inference that it *cannot* be the case that all interactions are governed by sonic Poincaré invariant laws.

Sound, with its direct theoretical *and* experimental binding to the medium of air, makes approach (c) above of eliminating the medium entirely untenable, at least when one extends the domain of inquiry beyond the ‘sonic laboratory’. As such, sound cannot qualify as fundamental, and is therefore inappropriate as an inductive base. On the other hand, there was for Einstein, and indeed still *is*, neither theoretical reason nor experimental evidence for implementing an ether in electromagnetism. In fact, Einstein’s radical act of eliminating the ether, which was a significant conceptual evolution beyond Poincaré and Lorentz, disconnected the effects of Poincaré invariance from any sort of matter or substance. Instead, he was arguing for a direct constraint on the electromagnetic field, now seen as fundamental, independent of possible idiosyncrasies arising from a medium. This legitimated the extrapolation to all fundamental fields the Poincaré symmetries as universal kinematic constraints. Transparently, sound is inappropriate for this role, given its inextricable ties to a medium.

Bringing these threads together, our point can be put like this. If a wave is taken to be non-fundamental in the sense that it is regarded as being an oscillation in some medium, then there must be some physical phenomenon that is a function of the state of that medium—for otherwise, one would have no empirical grounds for taking the wave to be non-fundamental. In turn, if there are physical phenomena that are functions of the state of the medium, then it cannot be the case that all physical fields are conditioned so as to obey the same symmetries as those of the wave under consideration—for otherwise, one would not observe these effects. Thus, if a wave is regarded as being non-fundamental, then one cannot thereby treat its symmetries as yielding a universal kinematical constraint on all fields. On the other hand, if a field is fundamental in the sense that it is regarded as being self-subsistent, rather than as being an oscillation in some medium, then there are no physical effects which are functions of the state of that putative medium, which means that one can legitimately make the inductive inference that all fields are conditioned so as to obey the symmetries associated with that field. Note, moreover, that this extrapolation is a necessary part of strategy (c) above. For example, in the case of the Michelson–Morley experiment, simply denying the existence of an ether is insufficient to account for the observed null results: one must in addition assume that the interferometer is *also* constructed from fields which obey optical Poincaré invariant laws—i.e., one must extrapolate the Poincaré symmetries of the electromagnetic equations governing light to physical fields including those fields out of which the interferometer is constructed.

If one prefers to avoid talk of fundamentality, then our point here could, alternatively, be put as follows: Einstein’s theory of special relativity, *qua* universal kinematical constraint, is to be understood as applying only to those fields for which we have independent reason to believe that the associated media do not exist. Since we *do* believe that the sound medium exists (it is, e.g., the air on Galileo’s ship), it is not appropriate to formulate this theory on the basis of a ‘sound postulate’, rather than a light postulate.^21^ That said, for those within the ‘sonic laboratory’, in which there is no empirical evidence for a sound medium as no known empirical phenomena depend on the velocity of the physical system under consideration with respect to that putative medium, option (c) above *would* become available, and—by exact analogy with the case of light—sonic relativity (featuring the sound postulate), understood as an inductive extrapolation of sonic Poincaré invariance, would be justified on empirical grounds (cf. [5, 61]).

## Gravitational Waves

Suppose, *per* the foregoing sections, that we take special relativity to be an inductive extrapolation of optical Poincaré invariance to all fundamental physical fields representing wave-like phenomena—i.e., to all such fields which we take to be self-subsistent, rather than to be oscillations in some medium.^22^ It is thereby reasonable to take these extrapolations to extend to other forces, such as the strong and weak nuclear forces—and, indeed, the extrapolation is successful, insofar as the fields associated with these forces are also governed by equations which are optical Poincaré invariant.^23^ A particular subtlety arises, however, in the case of gravity: should we infer from this extrapolation that gravitational waves in general relativity propagate at *c*? The answer to this question depends upon delicate interpretative matters regarding that theory.

As typically presented, the ontology of general relativity is represented by a Lorentzian metric field $g_{\mu \nu }$ on a differentiable manifold *M*, coupled via the Einstein equation to matter fields with stress-energy tensor $T_{\mu \nu }$. Gravitational waves are then taken to be wave-like oscillations in $g_{\mu \nu }$, induced by certain motions of bodies (e.g. infalling binary black holes, or neutron stars^24^). Understood in this way, gravitational waves are oscillations in $g_{\mu \nu }$—i.e., they have a medium, which is $g_{\mu \nu}.$ Thus, perhaps surprisingly, when understood in this way, the above inductive extrapolation should *not* apply to gravitational waves—i.e., we should not infer that gravitational waves propagate at *c*.

There is, however, another approach to understanding the ontology of general relativity. Typically, gravitational waves are modelled as perturbations $h_{\mu \nu }$ on a Minkowski background $\eta_{\mu \nu }$—that is, one has $g_{\mu \nu } = \eta_{\mu \nu } + h_{\mu \nu } + \mathcal{O}\left( h^2 \right)$. The so-called ‘spin-2’ approach to general relativity takes this picture seriously, postulating a flat Minkowksi spacetime $\eta_{\mu \nu }$ populated by a graviton field $h_{\mu \nu }$ (so-named for, on quantisation, such an object represents a spin-2 quantum field associated with gravitational effects). The graviton field obeys dynamics which can be shown to be equivalent to the Einstein equation.^25^ If one takes this ontological picture seriously, then gravitational waves should be associated with the propagating field $h_{\mu \nu }$; and now, in light of the self-subsistence and lack of medium of $h_{\mu \nu },$ the above extrapolation *should* apply—i.e., one should expect this wave to propagate at *c*.

In fact, however, one should not be too hasty in this reasoning. There is a rich history of debate in the foundations of gravitational wave theory regarding the extent to which the graviton field $h_{\mu \nu }$ is sufficiently mathematically and physically analogous to the electromagnetic field $A^\mu$ to warrant just such an extrapolation:^26^ luminaries such as Wheeler, Landau and Pirani accepted the analogy, whereas other equally eminent physicists such as Infeld, Havas, Eddington, Rosen, and Bondi rejected it [34, pp. 13–14]. (One might, indeed, take these concerns to be corroborated, insofar as recent work has indicated that the spin-2 field $h_{\mu \nu }$ need not invariably propagate at *c* [29].) For the purposes of this paper we of course do not need to resolve these debates. All we need point out is that the inductive extrapolation from the (optical) Poincaré invariance of the dynamical equations governing light to the dynamical equations governing gravitational waves only even potentially applies on one particular understanding of general relativity—namely, the spin-2 approach, on which the field $h_{\mu \nu }$ representing these gravitational waves is indeed construed as a sub-subsistent wave, rather than an oscillation in some medium.^27^

## Philosophical Upshots

We turn now to four foundational implications that arise from the above conclusions: implications regarding (i) emergent symmetries (Sect. 5.1), (ii) dynamical versus geometrical approaches to spacetime (Sect. 5.2), (iii) quantum gravity (Sect. 5.3), and (iv) spacetime functionalism (Sect. 5.4).

### Emergent Symmetries

In [65], Wallace points out that, starting from Saunders’ vector relationalism (set in Maxwellian spacetime) [56], there is a natural sense in which Galilean spacetime symmetries can emerge in a given subsystem. This is a case in which symmetries of a theory are *broken* in a subsystem: the latter are a proper subset of the former.^28^ As Wallace explains, “the distinction between ‘standard’ potential-based formulations of Newtonian gravity and the Newton–Cartan theory is just a matter of a preferred boundary condition” [65, p. 3].^29^ That is to say: this ‘scale-relativity’ of inertial structure is associated with the imposition of a preferred boundary condition upon a subsystem.

More specifically, Wallace takes a Newtonian theory set in Maxwellian spacetime, and considers a subsystem with either a finite number of particles, or, in the continuum case, finite mass (what he terms an ‘island universe’). He imposes a boundary condition on the gravitational potential *V*(*x*, *t*) associated with this subsystem, such that it obeys [65, p. 20]

2 $$\begin{aligned} \lim_{|x|\rightarrow \infty } V(x,t) = 0. \end{aligned}$$

The resulting spacetime recovers the Galilean symmetries: schematically, the additional degrees of freedom encoded in the boundary condition afford a notion of inertial structure. This breaks certain symmetries in Maxwellian spacetime, leading to the more restricted Galilean group. Thus, observers situated in this subsystem would be able to model the physics of their subsystem with Galilean invariant laws.

Sonic relativity illustrates an important foundational point: that the reverse case—subsystem symmetries being a proper superset (rather than a proper subset) of dynamical symmetries—can also arise. In this situation, when one considers the global physics, there is a preferred frame: the rest frame of the medium (thus, an appropriate spacetime setting globally could be Newtonian spacetime). However, an observer within a subsystem does not have access to this privileged frame: subsystem physics is decoupled from this global degree of freedom. That such physics is not sensitive to these degrees of freedom means that it is subject to fewer constraints, and thus that its symmetries can be richer than those of the global theory (in particular, physics within the subsystem might, as we have seen, be invariant under the Poincaré group, which is a *superset* of the global Newtonian symmetries^30^). As Todd et al. put the point, “...it can be seen that the existence of a preferred reference frame is not immediately prohibitive in the emergence of a self-consistent description of relativity by internal observers” [61, p. 1292].

Now, of course, to some extent this situation should be regarded as being an epistemological rather than a metaphysical issue: in the sonic relativity case, the preferred frame *still exists*—it is just that it is operationally irrelevant to observers within the subsystem. In terms of the physics which observers would be willing to construct and utilise, however (after following a fairly innocuous Occamist norm against the introduction of undetectable structure into one’s physics—cf. [19]), the following moral appears correct: *subsystem symmetries can be either a subset or a superset of global symmetries*.

Finally, we contend that one *can* construe along metaphysical lines our above point that subsystem symmetries can be a superset of global symmetries, if one is a certain stripe of ‘ontic structural realist’.^31^ For example, Ladyman and Ross argue in [37, Chap. 4] that ontological commitments in any particular domain of scientific inquiry are given by ‘real patterns’, which, roughly speaking, are empirical correlations which are (a) projectable, and (b) maximally information compressing.^32^ When one considers the physics within the ‘sonic laboratory’ in our above example, mention of the preferred global frame conveys no physically salient information; there are good grounds, therefore, to not regard it as part of the ontology within the laboratory; by contrast, the ‘real patterns’—and, therefore, the appropriate ontology for the special science of the sonic laboratory—will be cashed out in terms of sonic Lorentz invariant structures.

### Dynamical and Geometrical Approaches

Does spacetime structure explain the symmetries of the dynamical laws governing matter, or vice versa? This question lies at the centre of the well-known debate between ‘dynamical approaches’ to spacetime (which maintain that the dynamical laws explain spacetime structure: for the *locus classicus*, see [11]) and ‘geometrical approaches’ to spacetime (which maintain that spacetime structure explains the form of physical laws: for well-known defences, see e.g. [33, 42]). Proponents of the dynamical approach sometimes point to apparent problem cases for the opposing geometrical view, in which either (i) the laws governing matter do not manifest the same symmetries as the designated putative piece of spacetime structure (this is a case in which that latter structure fails to qualify as ‘theoretical spacetime’—see [52]), or (ii) the laws governing matter do manifest the same symmetries as the designated putative piece of spacetime structure, but nevertheless rods and clocks built from that matter do not survey that designated putative piece of spacetime structure (this is a case in which that latter structure fails to qualify as ‘operational spacetime’—see [51]). For an explicit presentation of such apparent problem cases, see [52]; for some nuancing of the issues, see [50].

We wish to become embroiled in these debates in this paper only to the extent that we wish to point out that the scenario of the ‘sonic laboratory’—in which the ‘true’ laws have a preferred rest frame (and so could be understood to have the symmetries of, say, Newtonian spacetime—see above), but in which the ‘emergent’ subsystem laws have a larger symmetry group (viz., at least the Poincaré group)—appears to be grist to the mill of the dynamical approach: for it is another illustration of phenomenological dynamical laws identifying correctly neither theoretical spacetime (for the Poincaré symmetries of the subsystem laws do not coincide with the Newtonian symmetries of the ‘true’ laws, associated with ‘true’ Newtonian spacetime structure) nor operational spacetime (for the rods and clocks constructed in the subsystems do not read off intervals of the ‘true’ spacetime structure—cf. again [61]).^33^^,^^34^

Nevertheless, proponents of the geometrical approach might not see the case of sonic relativity as being problematic for the view. After all, these transformations hold only within a certain subsystem. A geometrician could therefore argue that only *global* dynamical symmetries should be constrained by spacetime structure.

However, as [61] demonstrates, an observer within the system will not be able to detect any empirical effects in Galileo-ship type experiments within the medium (particle collisions and direct interactions with air particles notwithstanding), due to the effects of length contraction and time dilation. This would seem to suggest the possibility of returning to ether theory, given that we cannot determine whether we are encased in a similar subsystem of ether. As Brown states, “At the end of the day, it is always possible to add for whatever reason the notion of a privileged frame to special relativity, as long as one accepts that it will remain unobservable” [11, p. 67] (cf. [8, Sect. 4]). Given these concerns, the global status of the standard Poincaré transformations can be called into question: an issue which we explore further in the next subsection.

### Quantum Gravity

In Sect. 3, we argued that it was appropriate for Einstein to formulate his 1905 version of special relativity using the light postulate, but not the sound postulate, for light is a fundamental field, whereas sound is not, thereby legitimating the extrapolation to a ‘universal kinematical constraint’ in the former case, but not the latter. These arguments rested on a particular sense of ‘fundamental’—but there are, of course, others. In the sense that quantum electrodynamics (QED)—our current best theory of light—even *qua* theory of self-subsistent waves is expected to be reduced to some deeper theory of physics (whether that be electroweak theory, or a grand unified theory, or ultimately some theory of everything), it is *not* fundamental.^35^ But given this, one might maintain: just as agents in the ‘sonic laboratory’ might be led to believe that there is no preferred frame when in fact this does exist (the rest frame of the laboratory), so too might we be misled by our Poincaré invariant non-fundamental physics to believe that there is no preferred frame at the fundamental level.^36^ Given that QED is not fundamental (in this second sense), how do we know that we are in any better position that our ‘sonic experimentalists’?^37^

In our view, the above considerations do go some way to undercutting an insistence on Poincaré invariance when constructing theories of quantum gravity which could qualify as theories of everything—that is, when constructing physical theories which have the credentials to be truly fundamental. This reasoning should be congenial to authors who do, indeed, seek to break Poincaré invariance at the fundamental level: physicists such as Smolin [57] and Barbour [2, 3, 43] fall into this camp,^38^ as do philosophers such as Monton [44]. As Barceló and Jannes write,

The thought model that we have presented shows that, as long as there are no direct experimental constraints on this elementary description, one should take care when postulating which aspects of the currently known ‘effective’ physics (i.e. of the internal world) should also be taken as fundamental in the elementary description (the external world). [5, p. 198]

Although we concur that the above concerns are reasonable, we do, however, wish to emphasise one possible disanalogy between the parable of the ‘sonic laboratory’ on the one hand, and the current state of play in theoretical physics on the other. This is the following: in the former case, we were only motivated to think that there is indeed a preferred frame when considering a perspective external to the laboratory. However, no such considerations are at play in the quantum gravity case, as theories such as QED are understood to hold (at the appropriate energy levels) not just of subsystems, but of the entire universe. Thus, there is a disanalogy, for in the former case we are considering the phenomenological physics laws that apply in a given subsystem—what de Haro would call ‘extendable’ theories—while in the latter case we are considering theories which hold of the entire universe—what de Haro would call ‘unextendable’ theories [20]. It is not at all clear that the concerns that there could be additional spacetime structure, which proceed by considering some observer external to a given subsystem, apply in the latter case. This undercuts some of the force of the sonic relativity example. Of course, the sonic relativity case does serve to remind us of the long-appreciated possibility of global undetectable spacetime structure—such as the persisting points of absolute space in Newtonian spacetime, or even repostulating the ether as suggested above. However, as is also well-known, it is not at all clear that we should take such structure seriously, for it violates an Occamist norm (already mentioned in Sect. 5.1) to minimise one’s undetectable metaphysical commitments in one’s physical theorising.^39^

To summarise, then: there is a prima facie concern from sonic relativity that effective theories in some domain are compatible with base-level theories associated with a more structured spacetime. Insofar as theories such as QED are (just like sonic relativity) Poincaré invariant but non-fundamental (in the sense of this section), one might worry that our theories of quantum gravity need not, in fact, be Poincaré invariant, by analogy with the sonic relativity case.^40^ Although this is in principle correct, there is an important disanalogy, for theories of sonic relativity are theories holding only in some subsystem; by contrast, theories such as QED are supposed to obtain globally. Thus, concerns regarding the possibility of additional structure based upon considerations of subsystem-environment decompositions do not obviously apply in this case.

### Causality and Spacetime Functionalism

Suppose, hypothetically, that it were the case that the speed of sound *u* were greater than the speed of light *c*—in spite of the former propagating in a medium, and the latter being self-subsistent.^41^ Then, the limits of physical causality would be determined not by light, but rather by sound (assuming that there is no other faster wave). In such a scenario, it would still (as per Sect. 3) be inappropriate to use sound in a special relativistic theory, where that theory is construed as a kinematical constraint on fundamental fields (‘fundamental’ to be understood in the sense of Sect. 3). However, there is another sense in which it would indeed be appropriate to consider a theory based upon a relativity principle and a sound postulate: this would be a special relativistic theory construed as a theory delimiting the extent of the physically possible, given certain limitations on physical causality fixed by $u>c$.^42^

Now, we side with (at least the later) Einstein in understanding special relativity to be a kinematical constraint on fundamental fields (based upon an inductive extrapolation from the case of light).^43^ Although, in the actual world, certain reformulations of special relativity have been countenanced which seek to build up the entire content of this theory from constraints on causality (see e.g. [66]), it is worth noting that such interpretations of special relativity *qua* kinematical constraint would be inappropriate in worlds such as that countenanced above, in which these two senses of the theory diverge.

These considerations have a bearing upon the *en vogue* position of ‘spacetime functionalism’ (a term due to Knox [35], her own work growing out of that of Brown [11]), according to which “spacetime is as spacetime does” [38]. That is, spacetime is whatever plays the antecedently-delineated functional *role* of spacetime. For Knox, this role is to pick out a structure of local inertial frames (see [35] for a presentation of this view, and [51] for some concerns about the programme). For Baker, on the other hand, spacetime is a ‘cluster concept’: there are many different roles that one might expect spacetime to play—see [1, p. 14] for a (non-exhaustive) list. One such role might be to serve as a constraint on fundamental fields; another might be to delimit the extent of what is causally possible.

In worlds such as our own, these two criteria coincide—so both of the above spacetime roles would (as per the above) pick out the same spacetime structure. On the other hand, in worlds of the kind countenanced above in which the speed of sound exceeds the speed of light, the situation is more delicate—for one may obtain *different* verdicts upon what qualifies as spacetime, depending upon how one weighs these criteria. We do not intend this to be a critique of Baker’s approach to spacetime functionalism, but the matter does serve to reinforce the point made in [51, Sect. 6], that the approach is not as readily applicable to new cases as others, such as that of Knox.^44^

## Footnotes

1 In this paper, we take a ‘symmetry’ to be a transformation which leaves invariant the form of a given equation. For an excellent collection on foundational issues regarding symmetries, see [9].

2 (1) also has other symmetries: for example, it is invariant under conformal rescalings. Such additional symmetries will not matter for our purposes in this paper.

3 Of course, there are other differences between light and sound beyond symmetry considerations: for example, a sound wave can be represented by a scalar quantity $\psi$, whereas an electromagnetic wave is represented by a vector quantity $A^\mu$. The extent to which these mathematical differences are significant when drawing analogies between the physics of the propagation of such waves developed into a significant dispute in the context of gravitational wave physics—see [34], and Sect. 4.

4 See e.g. [58] for a history of these matters.

5 These articles are situated in the broader context of work on ‘analogue gravity’—i.e., the investigation of relativistic physics by way of surrogate physical systems. For comprehensive introductions to analogue gravity, see [6, 64]; for philosophical discussions regarding whether analogue gravity experiments can afford indirect empirical confirmation for predicted astronomical phenomena such as Hawking radiation, see [13, 15].

6 One might question the universal generalisation in our above parenthetical comment, ‘as with all waves’. It is possible that there do indeed wave-like phenomena whose velocities are functions of the speed of the source. For simplicity, however, we set aside such possibilities in the remainder of this article.

7 In fact, the ether hypothesis was made as early as 1690, in Huygens’ *Treatise on Light* [31]. For foundationally-oriented histories of ether hypotheses in the nineteenth century, see [11, 18, 54].

8 For an elegant mathematical result corroborating this claim, see [59].

9 See [11, Chap. 4] for an excellent history of the ether theorists and dynamical contraction hypotheses.

10 It is worth stressing again here that both light and sound are governed by a (Poincaré invariant) wave equation: in this sense, it is not light per se which caused such deep conceptual difficulties in the foundations of physics in the nineteenth century—rather, it was the expected non-motion of its *medium*, the ether, which caused these problems.

11 There is some subtlety to this issue, as many earlier ether theorists thought the ether to be non-fundamental and reducible to known matter: Fresnel and Cauchy both conceived of the ether as a molecular lattice, while British scientists such as Stokes and Green argued that the ether was a continuous ‘jelly’. Nevertheless, as Maxwellian electromagnetism and its description of light through electric and magnetic fields became widely accepted, the focus shifted away from characterising the mechanical details of the ether, opening the door for more novel conceptions of light and its medium. For example, FitzGerald believed that any comparison to normal matter would be inadequate, and subsequently conceived of the ether under his “vortex sponge” theory, completely distinct from other known matter. Later, Lorentz and Poincaré argued that the ether cannot be analysed in a mechanical way (e.g., on the model of a molecular lattice, or through particle collisions as with sound)—see [16, Sect. 9.2.4] and [30, p. 69]. For further discussion of different 19th Century physicists’ approach to the ether, see [16, Chap. 9] and [18, Chap. 6].

12 This conception of the equivalence principle was famously presented by Synge [60]; see [40] for discussion.

13 In our putting the views of the later Einstein in this way, we do not intend to adjudicate on the debate between dynamical and geometrical approaches to spacetime (see e.g. [11, 33] for two representative parties in this debate)—these matters are discussed in more detail in Sect. 5.

14 Although Einstein’s 1905 version of special relativity is often presented as following from the relativity principle, the light postulate, and certain auxiliary assumptions such as the homogeneity and isotropy of space and the ‘reciprocity’ principle (see [11, Sect. 5.4]), in our view there are yet more physical assumptions underlying this theory. First, one must assume that the inertial frames are global: see [52, Sect. 7]. Second (and more pertinent to our purposes), one must assume that the media of propagation of fields to which the theory is supposed to apply as a kinematical constraint (in the case of light, this medium being the luminiferious ether) in fact do not exist. It is for this reason that we take only option (c) above to lead to a ‘truly’ special relativistic theory. Our thanks to Adam Caulton for discussion on this point.

15 A Langevin clock consists of a wave bouncing between two mirrors, with a detector capable of registering a ‘click’ attached to one of the two mirrors.

16 Of course, in order for (c) to be a viable option here, the material out of which such clocks and other physical systems are constricted would also have to be governed by sonic Poincaré invariant laws: this point is stressed in e.g. [4, 5].

17 We consider in depth other senses of fundamentality in Sect. 5.3. Clearly, fundamentality in our sense is not a necessary condition for quantisability, given the possibility of, say, a quantum mechanical treatment of phonons: our thanks to Olivia Kelly for this point.

18 Cf. Kennefick: “...nowadays, insofar as we say that electromagnetic waves have a medium at all, we say that that medium is the electromagnetic field, an entity which is not even a part of the material world, although it is, of course, generated by particles that make up the material world.” [34, p. 5] (Note that Kennefick’s statement that electromagnetic fields are generated by particles does not imply the following more controversial claim: that electromagnetic fields are generated *exclusively* by particles. One might also question Kennefick’s claim that electromagnetic fields “are not part of the material world”—whether this is so will surely depend upon one’s understanding of ‘material’.)

19 Regardless of whether that ‘something’ is known matter, or is some entirely new class of substance—see footnote 11.

20 Recall from the previous paragraph that by a wave’s being self-subsistent we mean that it cannot be reduced to oscillations in some medium; this is consistent with the wave not being fundamental.

21 If one sought to generalise Einstein’s 1905 derivation, but not make reference to a *particular* invariant speed, one could, of course, appeal to the Ignatowkski transformations, which are derived without use of the light postulate. For discussion of von Ignatowski’s work, including references to original sources, see [11, 41, 63]. Given the non-fundamentality of sound waves and the existence of their medium, however, we would argue that it remains inappropriate to select an invariant speed from the Ignatowski transformations corresponding to the speed of sound.

22 By ‘optical Poincaré invariance’, we mean Poincaré invariance with invariant speed given by *c*. By ‘sonic Poincaré invariance’, we mean Poincaré invariance with invariant speed given by the speed of sound *u*.

23 See e.g. [47].

24 Note that we do not say ‘motions of *material* bodies’, as this would exclude by fiat black holes. For more on the conceptual and ontological status of black holes, see [14].

25 For recent detailed philosophical discussion of the spin-2 approach, see e.g. [48, 55]. These works include extensive references to the original physics literature on the subject. Note that the claim that spin-2 gravity is equivalent to general relativity is not entirely uncontroversial: see [46].

26 Cf. footnote 3.

27 Our thanks to Will Paisley and an anonymous referee for discussion on this section.

28 For a review of different spacetime settings for Newtonian mechanics, see [21, Chap. 2].

29 In [65], Wallace shows also that there is a precise sense in which Newton-Cartan theory is equivalent to Saunders’ vector relationalism: this reconciles the Wallace quotation with our above way of putting Wallace’s moral (which made reference to vector relationalism).

30 See [7]. To be completely clear here: by ‘global Newtonian symmetries’ we mean the symmetries of Newtonian spacetime—i.e., Galilean spacetime augmented with a standard of rest. These symmetries are associated with the ‘Newton group’: see [49, Sect. 3.1] for an explicit presentation of this group.

31 Structural realism in the philosophy of science was first presented by Worrall [67]; the distinction between ‘ontic’ and ‘epistemic’ structural realism was drawn by Ladyman in [36]. For recent book-length defences of ontic structural realism, see [25, 37].

32 Here is not the place for a detailed recapitulation of Ladyman and Ross’ ontic structuralist approach to ontology—for this, we refer the reader to [37].

33 There is a sense in which all of this would also hold in the hypothetical situation in which the ether theorists were correct: phenomenologically, the world would appear to be governed by Poincaré invariant laws, even though the ‘true’ laws would have different symmetries. The case of sonic relativity does, however, in our view, help to set these matters in new relief.

34 Even if the global symmetries were the Poincaré symmetries, there might still be a mismatch between the invariant speeds of the global versus subsystem symmetries: this would lead to the spacetime structure associated with the subsystem not being ‘cone-coincident theoretical spacetime’: see [51, Sect. 3.1].

35 Here, we set aside Cartwrightian concerns about this notion of fundamentality: see [12] for further discussion.

36 This preferred frame at the fundamental level need not be picked out via ‘surplus structure’, in the sense of [10].

37 A wave propagating in a medium would be reducible to (facts about) that medium—in which case, it could not be fundamental in the sense of this section. Thus, fundamentality in the sense of this section implies fundamentality in the sense of Sect. 3—but, of course, not *vice versa*.

38 These physicists offer different reasons to do away with Poincaré invariance: Smolin maintains that this is necessary in order to have laws of nature which can ‘evolve in time’; Barbour is motivated to this conclusion for reasons to do with spacetime relationalism.

39 That said, we see nothing wrong with the rejection of global Poincaré invariance as a means of guiding theory construction in the quest for a quantum theory of gravity: we thank an anonymous referee for stressing this point to us.

40 For discussion of the extent to which Poincaré invariance is present in a range of candidate quantum theories of gravity, see [39].

41 We make no claim that this situation ever arises in the actual world.

42 Cf. [53, pp. 128–129]. Of course, this hypothetical scenario could arise not just with sound, but any medium-dependent, non-fundamental wave with speed of propagation $u'>c$; the same considerations explained above regarding an extension to the limits of physical causality would apply when creating a ‘special relativistic’ theory based upon the speed of propagation of these waves.

43 Note again that we do not take this to commit us to a position in the debate regarding dynamical versus geometrical approaches to spacetime theories—cf. footnote 13.

44 Our thanks to Niels Linnemann for discussions on the content of this subsection.
